# Management factors affecting gestating sows’ welfare in group housing systems — A review

**DOI:** 10.5713/ab.22.0289

**Published:** 2022-10-11

**Authors:** Jae-Cheol Jang, Sang-Hyon Oh

**Affiliations:** 1 Department of Animal Science, College of Agriculture and Life Sciences, Gyeongsang National University, Jinju 52725, Korea

**Keywords:** Animal Welfare, Gestation Stalls, Housing Systems, Sows, Welfare Indicators

## Abstract

Public concern on the methods of raising food-producing animals has increased, especially in the last two decades, leading to voluntary and mandated changes in the animal production methods. The primary objective of these changes is to improve the welfare of farm animals. The use of gestational stalls is currently a major welfare issue in swine production. Several studies assessed the welfare of alternative housing systems for gestating sows. A comparative study was performed with gestating sows housed in either individual stalls or in groups in a pen with an electronic sow feeder. This review assessed the welfare of each housing system using physiological, behavioral, and reproductive performance criteria. The current review identified clear advantages and disadvantages of each housing system. Individual stall housing allowed each sow to be given an individually tailored diet without competition, but the sows had behavioral restrictions and showed stereotypical behaviors (e.g., bar biting, nosing, palate grinding, etc.). Group-housed sows had increased opportunities to display such behavior (e.g., ability to move around and social interactions); however, a higher prevalence of aggressive behavior, especially first mixing in static group type, caused a negative impact on longevity (more body lesions, scratch and bite injuries, and lameness, especially in subordinate sows). Conclusively, a more segmented and diversified welfare assessment could be beneficial for a precise evaluation of each housing system for sows. Further efforts should be made to reduce aggression-driven injuries and design housing systems (feeding regimen, floor, bedding, etc.) to improve the welfare of group-housed sows.

## INTRODUCTION

Animal production management has changed significantly across the European Union (EU) over the latter half of the 20th century [1]. During this period, pork production intensified, implying that the total number of breeding animals has increased, while the ratio of animal breeding farms has drastically decreased [2]. This phenomenon appears to be strongly related to increased household income. With global economic development, per capita income has grown rapidly, leading to significant changes in the patterns of food consumption—from grains to livestock-derived products [3]. To meet consumer demand, large numbers of animals are moved to indoor housing systems with lower space allowances, and the use of prophylactic medicines and growth promoters has increased [4]. This intensification of the industry increased productivity but decreased the monetary value of any given animal [5]. Widespread concern about farm animal welfare was highlighted in response to this campaign because evidence showed that keeping farm animals in intensive conditions may lead to a reduction in the welfare status of animals [6]. Pigs (Sus scofa domesticus) are the most intensively reared mammals in the world [7], with approximately 1.3 billion pigs slaughtered annually for meat. Although legislation to optimize conditions for the protection of pigs has recently surpassed that required by EU law (e.g., Animal Welfare Act 2006), it does not solve all the welfare concerns associated with conventional pig rearing [1].

Several studies suggested ways to protect livestock against confinement conditions. In gestating sows, individual stall management is widely used for the ease of artificial insemination, low capital cost, and minimization of overt aggressive behaviors [8]. However, the restriction of movement, impossibility of performing normal feeding, and disruptive patterns of behavior cause welfare problems, such as the development of stereotypes, chronic stress, lameness, and decubital ulcers [9]. Several studies compared the different indicators of welfare and productivity in stalls and in modern commercial group housing systems. Group-housed sows with an electronic sow feeding (ESF) system have similar or improved productivity compared with sows housed in stalls [10]. Furthermore, no differences in stress-related serum cortisol concentrations were evident between sows housed in stalls and those housed in groups [11,12].

This review explores the definition of animal welfare, parameters for welfare assessment, and rapidly accumulating data concerning the impact of the housing systems on welfare in gestating sows. The main factor affecting welfare issues include group housing for gestating sows. Welfare during the other phases of pig production (nursery, growing-finishing pigs, and farrowing sows) is outside the scope of this review

## DEFINITION OF WELFARE

Animal welfare started with the publication of the Brambell report on the welfare of farm animals, issued by the World Organisation for Animal Health (formerly the Office International des Epizooties; OIE) in 1965 [13]. In the report, animal welfare is defined as “the physical and mental state of an animal in relation to the conditions in which it lives and dies” [13]. Since then, considerable research has been conducted on animal welfare problems involving scientific fields of interest, such as the development of welfare assessment in various environmental conditions, as well as on more fundamental questions linking the biological bases of welfare and stress [14]. Freedom plays a key role in animal husbandry. In fact, the Farm Animal Welfare Council defined knowledge about the needs of animals, which is related to the proposal to give animals some freedom (Table 1).

According to the study of Carenzi and Verga [14], there are three aspects of welfare evaluation. The first approach emphasizes the biological functions of the organism, such as growth and reproductive performance, as well as its health status and behavioral characteristics. Behavior reflects the foremost response to environmental stimuli and may provide a clear signal of stressors. Qualitative welfare levels reflect the absence of distress or a strong stress response [15]. A second approach suggests that the relationship between stress and welfare stress in terms of psychological aspects, considering feelings as a key element in determining quality of life. The third approach emphasizes natural living, insisting that animals should be allowed to live according to their natural attitudes and behaviors, mainly developing and using their natural adaptations. However, due to the domestication process, domestic animals differ in many ways from their co-specifics, and it is difficult to assess welfare levels in a scientific way. A more comprehensive approach to animal welfare, categorized into four main issues, was proposed by Dockès and Kling-Eveillard [16]:

i) Biological and technical definitions stress the fundamental needs of animals and their freedom, as well as the possibilities to cope with environmental challenges.
ii) Regulation approaches, which recognize the animal as a sensitive being and as such must be put in conditions “compatible with the biological needs of the species.”
iii) Philosophical approaches, which consider the “status of the animal” and its role in human society.
vi) Communication between humans and animals is of great importance to the farmer-animal interaction and its effects on industrial breeding systems.

## ASSESSMENT OF ANIMAL WELFARE

Welfare criteria is a multifactorial concept that relies on the analysis of the interaction between animals and their environment, which include behavior and the biology between the hypothalamic-pituitary-adrenocortical (HPA) axis and the autonomic nervous system (ANS), as well as animal’s consequences on production traits and possibly the health status [17]. This is the reason the welfare categories are not uniformly agreed upon by various stakeholders, including scientists, producers, and consumer protection unions. Based on this standard, welfare methods can be categorized into three types: animal-, resource-, and management-based. Welfare Quality is one of the most widespread animal welfare assessment protocols for animal-based indicators, including lameness, body condition score, qualitative behavior assessment, and the human-animal relationship test [18]. In this review, we focus on welfare assessment based on resource indicators, as followed by McGlone et al [19].

### Physiology

Various biological systems, such as the cardiovascular system, gastrointestinal system, exocrine system, and adrenal medulla, are controlled and influenced by the ANS during stress [20]. However, it is controversial whether stress activation of the ANS significantly affects the long-term welfare of an animal, owing to the relatively short duration of the biological effect on autonomic responses [21]. In fact, the plasma levels of catecholamines are extremely sensitive to handling, and hence, more surgical blood sampling methods, such as direct venous puncture or chronic catheter, must be considered [22]. Fernández et al [23] suggested that this short-term acute response can be diagnosed using various measurements, such as heart rate, blood pressure, plasma glucose, and fatty acid levels. Furthermore, the value of monitoring ANS activity is subject to various factors, such as locomotion, physical activity, and/or feed intake [24]. The concentrations of plasma glucose and fatty acids represent the energy balance between the mobilization of energy stores and use of energetic metabolites, whereas the concentration of serum lactic acid reflects anaerobic metabolism [25]. These metabolic measurements are frequently connected with assays for the determination of circulating enzyme activity, such as transaminases and creatine kinase, which are widely used to detect susceptibility to stress in pigs [26].

**Figure 1:**
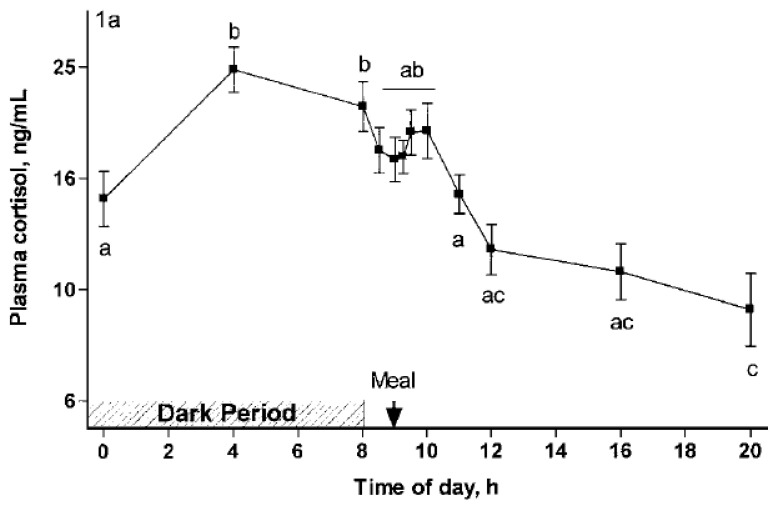
Diurnal changes in plasma cortisol of gestating sows fitted with an indwelling jugular catheter. The meal-induced release of cortisol is clearly visible [32].

Contrastingly, HPA activity, with the release of cortisol, a cholesterol-derived steroid synthesized in the fascicular zone of the adrenal cortex under the control of the pituitary hormone adrenocorticotropic hormone, released in the general circulation to reach its receptors in tissues, has a broad and long-lasting effect on the body [27]. Cortisol exhibits catabolic activity in peripheral tissues and anabolic activity in the liver, including gluconeogenesis and protein synthesis [28]. Since cortisol also reduces the entrance of glucose into cells, it increases blood glucose and insulin secretion, resulting in the storage of energy as fat in adipose tissue. Consequently, this increases fat deposits at the expense of tissue proteins [29]. Furthermore, cortisol increases appetite by stimulating the arcuate and ventromedial hypothalamus in the brain [30]. This is frequently the case in homeostatic regulations; the increase in energy availability is a coordinated process via peripheral and central mechanisms [31]. Although the features of the HPA axis are not specifically documented in pigs, cortisol is highly susceptible to a diurnal cycle that is genetically determined by light and feed intake [32] (Figure 1).

### Behavior

Behavior is the primary way of interaction; therefore, it can be a sensitive indicator of the animal’s perception of environmental changes [33]. Various behavioral patterns often reflect the first level of response of an animal to a stressful environment. Thus, behavior is used extensively to analyze environmental needs and preferences [34]. Additionally, it is also a classical symptom in the examination of health problems, such as general behavioral depression accompanying fever, known as sickness behavior, or lameness indicative of locomotor problems [35].

In addition to sickness-related behavior, other changes in the duration and frequency of normal behavior are recognized as indicators of mental suffering [36]. Furthermore, Cook et al [36] noted that there are numerous possible signs of stress, including startle or defense response, avoidance, excessive aggression, stereotypic behavior, and lack of responsiveness. Although not all these various behaviors are signs of poor welfare, they can be a warning sign if accompanied by other symptoms [37]. Redirected injurious behaviors, such as tail biting in pigs, correlated with a lack of exploratory activity, are abnormal behaviors that may easily lead to pain. Thus, acceptance of the behavioral need for this exploration, as well as frequencies of redirected behaviors, can be used as indicators of welfare [38,39].

Aggressive behaviors are major expressions of the social interaction of pigs [40]. The most aggressive behavior appears in relation to feed competition or mixing [41–43]. Such behavior can be observed in intensive commercial pig housing systems, especially when unknown pigs are mixed into new groups [44]. Aggressive behavior occurs in several circumstances, such as body weight difference, various space or group sizes, or familiarity [33,45]. Aggressive encounters often result in skin injury and can have immunosuppressive effects [46].

An animal displaying stereotypical behavior repeats a relatively invariant sequence of behaviors that has a purposeless function [47]. The impossibility of displaying a behavioral need can also lead to the appearance of stereotypies. Various abnormal behaviors occur in farm animals, including bar biting in confined sows, tongue rolling in cows, and crib-biting in horses [48].

### Performance

Although the relationship between production and welfare is not simple and difficult to interpret, performance parameters provide an overview of the problems that reflect optimum welfare [49,50]. Practices to improve production via the use of growth promoters are questioned because they may have a detrimental impact on welfare or mask the negative impact or poor welfare on production performance [51]. Therefore, it is better to approach the welfare assessment of performance in terms of health status rather than productivity. Representative parameters include mortality in growing-finishing pigs and reproductive performance of sows (stillborn, mummy, weaned pigs, culling rate, farrowing rate, and weaning to estrus interval). Mortality rate is influenced by various factors, such as housing conditions, management, group size, and stockmanship [52]. In sows, poor reproductive performance may be related to stress. A possible explanation reported by Wan et al [53] is that glucocorticoid hormones reduce the activity of sex neuroendocrine systems and therefore reduce the efficiency of reproductive performance. In finishing pigs, agonistic productivity can have detrimental effects on welfare and reduce weight gain [54]. They also compromise pork quality, giving pork low pH and pale, soft, and exudative, which reflects the economic crisis underlying pig production [55].

## GROUP HOUSING OF GESTATING SOWS

A major public concern regarding farm animal welfare is focused on gestating sows (Council Directive 91/630/EEC, 1991). Under commercial conditions, gestating sows are predominantly accommodated in gestation stalls, which are both physically and psychologically detrimental to sows [19,56]. In fact, much compelling evidence exists that the European Union’s (EU) Agriculture Council, which consists of agriculture ministers from 15 member countries of the EU, issued a directive addressing gestation stalls (Council Directive 2001/88/EC, herein referred to as the “EU Pigs Directive”) that will apply to newly built facilities as of 2003 and all other facilities as of 2013. The directive bans the use of stalls after the fourth week of pregnancy and tethers completely. Nevertheless, the major pork-producing countries (e.g., East and South-East Asia, USA, and South America) still use stall housing because of the ease of artificial insemination, low capital cost, individual feeding, and minimization of aggressive behavior [8]. However, stall housing has a negative effect on muscle weight and bone strength [57], decubital ulcers, chronic diseases, and stereotypes, which indicates poor welfare of sows [9].

There are advantages and disadvantages to group housing and individual stall systems. Individual stalls can reduce labor costs, are more manageable, have earlier morbidity detection, and can control feed intake [58]. Additionally, the stall protects the sow from aggressive encounters that normally occur during the regrouping of sows in group pens, which occur throughout a sow’s lifetime [59]. Contrastingly, the major difference between the group housing system and stall management is that the former provides freedom of movement by providing enough space to turn around, lie down, stand up, stretch limbs, and groom [60]. This is commonly known as dynamic space, or the space necessary to make postural adjustments or turn around [59]. Restricting the ability of the sow to walk and turn around may affect their health, performance, and overall wellbeing [58,60].

## FACTORS AFFECTING THE WELFARE OF GROUP HOUSING SYSTEM

### Space allowance

The minimum space requirement for a sow in group housing remains controversial. The European Food Safety Authority [61] describes the three types of space required to aid the estimation of the levels of space required by pigs: static, behavioral, and interaction space. The static space required for pigs to lie, or stand can be calculated using the equation A = *k*×W^0.666^, where A is the area in m^2^, W is body weight in kg, and k is a constant depending on the posture of the animal. Examples of *k* = 0.019 for sternal lying (and standing) and *k* = 0.047 for fully recumbent pigs [62].

Many scientific studies related to space allowance in pigs measured the occurrence of aggressive interactions as an important outcome of feeding system types [63–66]. They concluded that the effect of floor space on aggression particularly increased early after mixing. In gilts, Barnett et al [63] observed that on days 2 to 54 after mixing, increasing space reduced aggressive behaviors, such as bites and butts. Similarly, the number of threats, withdrawals, and head interactions, including bite and nose interactions, were reduced with increasing space on days 6 and 7 after mixing in sows [64]. Furthermore, Remience et al [66] found that nonreciprocal aggression on days 3 and 8 after mixing was greater in pregnant sows in a smaller floor space, although reciprocal aggressive behavior (bites or knocks) did not differ. For sows mixed soon after insemination, increasing space reduced feeding aggression on day 2 after mixing, but not on day 8 [67].

Increased aggressive behavior was correlated with decreased space allowance. Weng et al [64] reported that more injuries were observed with greater space restrictions in the group housing system. Similarly, Remience et al [66] noted that more fresh superficial injuries and deep skin injuries were reported when less space (2.25 versus 3.0 m2/sow) was provided in ESF group housing. Furthermore, Salak-Johnson et al [65] stated that skin injuries increased as floor space decreased. However, Hemsworth et al [67] concluded that although space affected aggression and stress, it did not affect skin injuries. These conflicting results may be due to different experimental conditions, such as floor feeding, ESF, and static and dynamic groups.

The immune system is one of the mechanisms developed by organisms to defend against environmental challenges and other perceived threats [68]. Several studies concluded that chronic stress exerts a general immunosuppressive effect that suppresses or withholds the ability of the body to initiate a prompt and efficient immune reaction [69]. This is due to high levels of corticosteroid production during chronic stress, which produces an imbalance in corticosteroid levels [70]. Plasma cortisol levels and changes in leukocyte populations are the most common physiological parameters used to measure farm animal welfare [19]. A study by Salak-Johnson et al [68] found differences in cortisol, neutrophil, and lymphocyte populations, and neutrophil-to-lymphocyte (N:L) ratio, with sows housed at the greatest floor space allowance having the lowest N:L ratio but the highest plasma cortisol. Contrastingly, most studies found no difference in plasma cortisol [12,71] or immune activity, more specifically N:L ratio [12,72] among sows housed in stalls or pens.

### Group size

Group size is defined by the number of sows in a pen rather than by the amount of space allotted to each sow [73]. It was reported earlier that aggression increases in large groups due to the establishment of a dominance hierarchy [34,74]. However, recent reviews concluded that there is no evidence to suggest that there is more aggression in large groups of up to 40 and 300 sows in experimental settings and commercial conditions, respectively [58,75]. This also supports the findings of Hemsworth et al [67], who indicated that there was no statistical difference in the frequency of aggression in group-housing sows in the early gestation period (days 2 and 8 after mixing) into groups of 10, 30, and 80. Therefore, the author speculated that group size is correlated with the ability to socialize sows [67]. In large groups, where individual recognition is less likely, animals use methods other than aggression to establish social dominance, such as body size [76]. Group size had no effect on reproductive performance [67,74], as well as serum cortisol concentration [67]. Anil et al [77] noted that sows exposed to the aggression associated with mixing and the ESF before implantation may have a lack of difference in reproductive performance between the different size groups.

### Group type

Under commercial conditions, gestating sows can be managed in either static or dynamic groups. For the static groups, all sows in a group were introduced on the same day and remained until the entire group was moved to the farrowing facility. Static grouping involves forming a pen group once without adding more females after the group is established. In dynamic groups, small groups of sows are added to a larger existing group periodically throughout gestation, and groups are removed periodically as sows move farrowing. A new bout of aggression occurs each time a new group is added [78]. However, sows in large dynamic groups are shown to adopt a more tolerant and passive response to unfamiliar animals [79].

Although few studies pointed out that greater aggressive behavior originates from frequent mixing in dynamic groups [34,58,80], other findings do not support this interpretation. According to a study by Van der Mheen et al [81], sows in large dynamic groups (50 sows) consumed their individual ration in smaller portions due to disturbances in the feeders compared to that in small static groups (13 sows). Additionally, these authors found that sows in dynamic groups recorded higher incidences of skin scratches, but no differences between treatments were observed in pregnancy rates, litter size, or litter weight [81]. In agreement with this, Anil et al [82] found that although skin injury scores were greatest in the dynamic group both in general and 2 weeks after mixing, there were no effects on aggression, cortisol concentrations, farrowing performance, and longevity. Furthermore, a study by Strawford et al [83] found no differences in aggression, skin injuries, and cortisol concentrations between sows in static and dynamic groups with ESF.

### Feeding regime

It is considered that a restricted amount of feed is commonly provided to breeding sows to prevent excess BW gain and fat deposition, which can cause farrowing and locomotion problems and subsequently reduce reproductive performance [84]. In the pork industry, it is generally considered that a restricted level of feeding during gestation is sufficient for maintenance and fetal development, suggesting that animals do not have a negative energy balance [85]. However, limited feeding results in more competition for feed or access to feeding areas, and the development of stereotypies [58]. In the condition of group housing, there is no clear evidence in the literature on increased aggression, stress, or injuries associated with restricted feeding levels. According to a study by Spoolder at al [86], although there were no effects on aggression or skin injuries, grouped sows fed 1.8 kg (23 MJ digestible energy (DE)/d) in “lock-in” stalls spent more time standing and manipulating bars and chains after feeding than those that were fed 3.2 kg (40 MJ DE/d). Bergeron and Gonyou [87] found that sows fed either a high-energy diet (23.7 MJ DE/kg) or a “high-foraging” diet (a standard diet [14.0 MJ DE/kg] but with a device in the feeder that increased the feeding time) spent less time active and displaying stereotypies than sows fed with a standard diet (14.0 MJ DE/kg). Therefore, a lack of energy from diet and time spent feeding may contribute to the development of stereotypies [85]. However, although increased feeding times are shown to reduce sow hunger, sequential feeding systems such as ESF can cause crowding, thereby reducing the overall feeder capacity [88].

The type of feeding system affects the level of aggression related to feed competition [75]. There are three representative feeding types in the group-housing system: floor feeding, partial stalls, and ESF. Floor feeding is the simplest and cheapest among the systems. This system allows sows to feed simultaneously, and thus fulfills some elements of natural feeding behavior. However, variation in feed consumption between dominant and subordinate sows is also observed in floor feeding systems, causing subordinates to suffer from undernourishment and low weight gain [89]. Contrastingly, partial stall reduces aggression and plasma cortisol concentrations in the long term in group-housed gestating gilts [90,91]. Most welfare concerns in this system are the incidence of vulvar biting. Andersen et al [91] found that sows housed in pens with full-body feeding stalls had increased vulva bites and suggested that feeding arrangement influences nature as well as the amount of aggression. Although floor feeding is competitive, gaining access to feeding stalls can also lead to competition and aggression between group-housed sows [73]. The most advantageous group housing system that deals with the individual feed consumption of sows is the ESF. This allowed for the greatest possible control over individual sow intake. However, this system forces the sows to feed in sequence, and as such, the sows queue at the ESF entrance gate. These findings support recent work by Olsson et al [92], who observed that approximately 4 to 6 sows often queue at the ESF entrance, although one-third of queued sows have already eaten daily feed rations. Consequently, preventing queuing was identified as an important development for improving welfare in ESF systems [82].

### Bedding

Although the influence of bedding quality on welfare, health, and performance of animals is not extensively studied, the most common enrichment and bedding materials for group-housed sows reported in the literature are straw [93] or rice hulls [94]. In fact, straw offers excellent possibilities for diverse manipulations: to root or scratch in, to chew, and to eat. Andersen et al [91] found that in group-housed sows, the supply of a bedding substrate reduced the frequency of abnormal gait compared to sows raised on a slatted floor. Beddings also play an important role in group housing designs, as they absorb excreta and are used to enhance the thermoregulatory abilities in sows [95]. Therefore, group housing with straw bedding is almost always associated with large dynamic groups and ESF feeding [75]. This suggests that in large groups with a tendency for higher incidences of aggression, enrichment and bedding may be an effective means of improving sow welfare [73]. However, the use of straw is not without its disadvantages, mainly due to cost, increased labor, hygiene concerns, and most importantly, incompatibility with manure and drainage systems [96]. Bench et al [88] stated that several factors make it difficult to evaluate the welfare relevance of straw from the scientific literature: i) the variation in the composition, structure, quality, and quantity of straw; ii) no scientifically authorized or qualified assessment of animal welfare on the effect of straw; iii) lack of specific investigation on the welfare impact of straw; and v) the importance of straw with the age of the animal and their housing conditions and management.

## CONCLUSION

In this review, the advantages and disadvantages of welfare indicators are highlighted and broadly discussed. For gestating sows, group-housed sows negatively influence aggressive behavior during the establishment of a social hierarchy, causing a negative impact on longevity (more body lesions and lameness). Sow housing in individual stalls leads to more stereotypical behaviors. However, there were inconsistent results on reproductive performance across studies. More validated and reliable resource-based sow welfare assessment protocols should be developed with the help of farmers, experts, stakeholders, and consumers.

## Figures and Tables

**Table 1:** The five freedoms as the fundamental experience goals for animals

Freedom	How
1. Freedom from hunger and thirst	By ready access to fresh water and a diet to maintain full health and behavior.
2. Freedom from discomfort	By providing an appropriate environment including shelter and a comfortable resting area.
3. Freedom from pain, injury, or disease	By prevention or rapid diagnosis and treatment
4. Freedom to express normal behavior	By providing sufficient space, proper facilities, and a company of the animal’s own kind. Also: Possibility to carry out natural behaviors.
5. Freedom from fear and distress	By ensuring conditions and treatment which avoid mental suffering.

Farm Animal Welfare Council [13].
